# *In vivo* articular cartilage deformation: noninvasive quantification of intratissue strain during joint contact in the human knee

**DOI:** 10.1038/srep19220

**Published:** 2016-01-11

**Authors:** Deva D. Chan, Luyao Cai, Kent D. Butz, Stephen B. Trippel, Eric A. Nauman, Corey P. Neu

**Affiliations:** 1 Weldon School of Biomedical Engineering, Purdue University, West Lafayette, IN, 47907; 2 School of Mechanical Engineering, Purdue University, West Lafayette, IN, 47907; 3 Departments of Orthopaedic Surgery and Department of Anatomy and Cell Biology, Indiana University School of Medicine, Indianapolis, IN, 46202; 4 Department of Mechanical Engineering, University of Colorado, Boulder, CO, 80309.

## Abstract

The *in vivo* measurement of articular cartilage deformation is essential to understand how mechanical forces distribute throughout the healthy tissue and change over time in the pathologic joint. Displacements or strain may serve as a functional imaging biomarker for healthy, diseased, and repaired tissues, but unfortunately intratissue cartilage deformation *in vivo* is largely unknown. Here, we directly quantified for the first time deformation patterns through the thickness of tibiofemoral articular cartilage in healthy human volunteers. Magnetic resonance imaging acquisitions were synchronized with physiologically relevant compressive loading and used to visualize and measure regional displacement and strain of tibiofemoral articular cartilage in a sagittal plane. We found that compression (of 1/2 body weight) applied at the foot produced a sliding, rigid-body displacement at the tibiofemoral cartilage interface, that loading generated subject- and gender-specific and regionally complex patterns of intratissue strains, and that dominant cartilage strains (approaching 12%) were in shear. Maximum principle and shear strain measures in the tibia were correlated with body mass index. Our MRI-based approach may accelerate the development of regenerative therapies for diseased or damaged cartilage, which is currently limited by the lack of reliable *in vivo* methods for noninvasive assessment of functional changes following treatment.

Noninvasive visualization and quantification of *in vivo* anatomy and physiology has long been a goal of medicine and engineering. The ability to measure biomechanical quantities in biological systems that are designed to generate, transmit, or receive loads could greatly enhance knowledge and understanding of tissue health, damage, disease, and repair. The living human body poses an exquisite mechanical environment, resulting in complex physical interactions that cannot be fully understood with *ex vivo* experiments alone. Especially in tissues with a primarily mechanical function, such as articular cartilage, knowledge of the localized micromechanical environment can lead to a further understanding of mechanobiology and the biomechanical response of healthy and pathologic tissues^1^,^2^. Additionally, biomechanics of tissues, elucidated by noninvasive imaging, can serve as a unique biomarker for disease monitoring and tissue engineering^3^ and a critical source for *in vivo* experimental data in the validation of constitutive relationships^4^.

Despite the importance of mechanical function to the physiology of connective tissues, the visualization of the *in situ* mechanical behavior of orthopaedic tissues in particular has been technical challenging^5^,^6^, and methods for the direct, noninvasive *in vivo* measurement of internal cartilage mechanics (i.e., strains *within* the tissue) are completely lacking^7^. Although rigid body motion of connective and orthopaedic tissues, e.g. bone, can be easily assessed using videographic or fluoroscopic motion analysis^8^,^9^ and contact pressures can be measured using telemetric joint implants^10^,^11^, internal tissue deformations are challenging to visualize due to a number of inherent obstacles. Orthopaedic tissues are stiffer than many other soft tissues in the body, leading to small deformations that are difficult to measure noninvasively and require high spatial resolution. The hierarchical anatomy of most orthopaedic tissues^12^ means that, for a comprehensive understanding of the mechanics, tissue behavior should be examined at several length scales, either through direct measurement or mathematical modeling based on direct measurements. These and other difficulties have led most researchers to concentrate on characterizing the mechanics of orthopaedic tissues such as articular cartilage in controlled laboratory environments, often as tissue cultures or explants.

Noninvasive measurement and estimation of the mechanical behavior of articular cartilage has been approached using various techniques and at different length scales in living humans (Fig. 1). For tissue-level studies, joints can be imaged *in vivo* for tissue morphology, using magnetic resonance imaging (MRI) or other high-resolution techniques, and kinematic (i.e. velocity) data for joint segments can be assessed via videographic, fluoroscopic, or MRI-based motion analysis^9^,^13^,^14^. Coupled with kinetic measures (i.e. ground reaction forces) and other externally obtainable measures (e.g. electromyography), mathematical models can be developed to estimate the mechanical behavior at the tissue level^15^,^16^,^17^,^18^. Alternatively, specific exercises or loading regimes to target the deformation of a particular tissue can be performed before or during imaging with MRI, computed tomography, or other techniques to measure the load response of tissue^19^,^20^,^21^,^22^. At levels smaller than tissue level though, noninvasive imaging techniques are still being developed^23^, and techniques in biophotonics may be needed for study of *in vivo* cellular and subcellular biomechanics.

Few noninvasive techniques are designed to be able to measure internal mechanical behavior, which can vary by location and length scale, especially in complex and hierarchical biomaterials that exhibit heterogeneity, viscoelasticity, and anisotropy. Although based on noninvasive morphologic imaging, mathematical models of *in vivo* joint biomechanics often rely on various assumptions of material properties or tissue behavior to estimate internal tissue deformations^16^,^24^,^25^ and do not directly measure the actual mechanical behavior of tissue. Magnetic resonance elastography (MRE) can be used to estimate the stiffness of softer biomaterials, typically under high frequency loading;^26^ however, MRE in orthopaedic soft tissues has been restricted to *ex vivo* applications^27^,^28^. For *in situ* and especially *in vivo* MRE of orthopaedic tissues, the technical challenge of propagating high frequency compression or shear waves to deeply embedded tissues is compounded by the relatively short length scale (i.e. thickness) and high stiffness of tissues such as articular cartilage. The applicability of high frequency, low magnitude loading to viscoelastic tissues like cartilage, which often encounter only low frequency, large magnitude loads in normal *in vivo* function, is also debatable. On the other hand, techniques that estimate deformation fields during static load using texture correlation^29^ or measure pixel displacements during low frequency loading using displacement-encoded MRI^30^ can directly measure *in situ* tissue strains on a clinical MRI system. These approaches show promise for the measurement of *in vivo* biomechanical behavior. It is important to note though that the relative stiffness and thinness of articular cartilage borders on the technical limits of current clinical MRI, even for phase contrast MRI techniques that are compatible with low frequency loading, such as the one used in this study.

Despite the difficulties of *in vivo* measurements, the importance of mechanical stimuli to the physiology of living articular cartilage is clear^31^. In addition, the rising recognition of the importance of mechanobiology to cartilage physiology at cell and tissue levels^32^ further warrants the focus on noninvasive measurement of tissue mechanics *in vivo*. Although tissue morphology can be visualized before and during cyclic loading to extrapolate mechanical behavior, measurement of changes to nominal thickness or volume do not reveal internal mechanical behaviors of mechanically heterogeneous tissues. For this study, noninvasive measurement of displacements under applied loading with MRI (dualMRI), which takes advantage of synchronized and combined (i.e. dual) MRI and loading apparatus actions, was chosen because displacement-encoded MRI has been shown to be able to measure intratissue displacements and strains at high precision^33^,^34^.

We quantified the intratissue mechanical behavior of tibiofemoral articular cartilage in a sagittal plane through the knees of nine volunteers with dualMRI. We hypothesized that compressive loading applied to the foot, and the resulting contact in the tibiofemoral joint (i.e. knee), would give rise to complex patterns of cartilage strain characterized by high shear magnitudes. We further hypothesized that male and female subjects would exhibit similar patterns and magnitudes of cartilage strain. To address these hypotheses, synchronization of displacement-encoded MRI to cyclical loading of the leg, generated by an MRI-compatible loading device, enabled the visualization of soft tissue motion and contact. Phase data from dualMRI allowed for quantification of intratissue deformation, in other words the measurement of displacement at each imaged pixel/voxel, during cyclic loading. To study the relationship between strain measured in this system and body mass index and gender, the maximum values for shear and principal strains of each subject were further analyzed. Characterization of *in vivo* mechanics in tibiofemoral cartilage as a model system demonstrates the utility and broad applicability of dualMRI for the study of mechanical behavior in a range of orthopaedic soft tissues.

## Results

Institutional Review Board approval was obtained for this study. *In vivo* displacements and strains were quantified throughout the volume of the tibiofemoral cartilage of healthy human subjects using dualMRI (see *Methods*). An MRI-compatible pneumatic loading device was tested prior to imaging to show that legs of living subjects could achieve a quasi-steady state response to loading, required to approximate a slow walking cadence at physiologically relevant load ranges as well as minimize motion artifacts during imaging. Parameters for *in vivo* imaging were selected based on displacement and strain precisions measured using an imaging phantom. Analysis of dualMRI data then permitted the estimation of *in vivo* strains in regions of interest (ROIs) defined in tibial and femoral cartilage of healthy human subjects.

### Quasi-steady State Load Response

**Figure 2:**
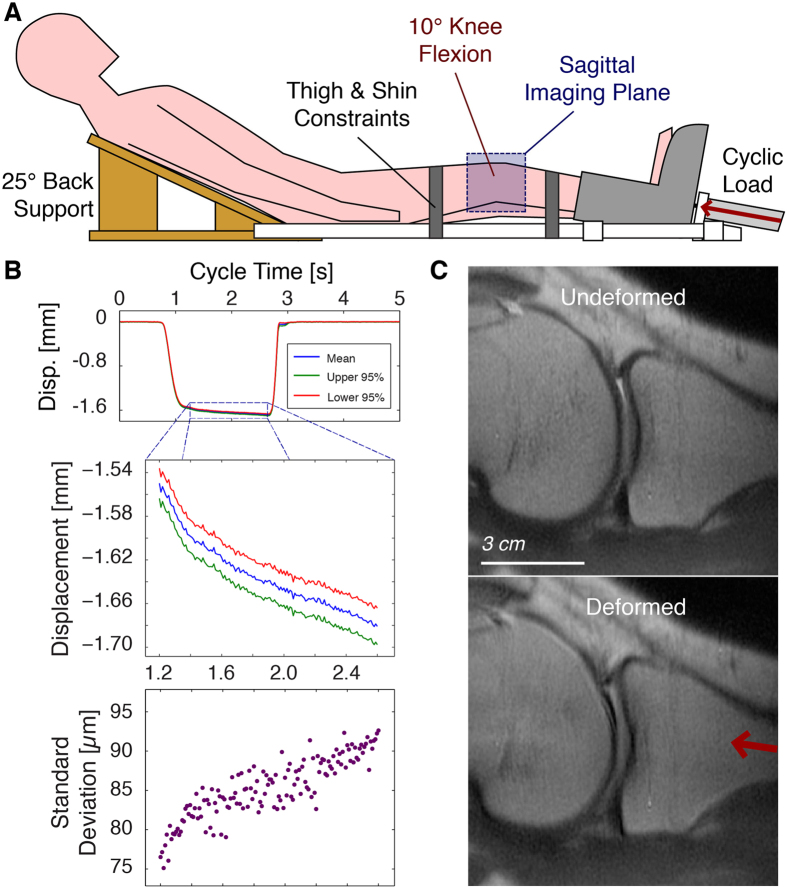
MRI-compatible knee loading device for *in vivo* measurement of tissue deformation. A loading device was manufactured to permit the cyclic loading of the leg of a human subject by a pneumatic actuator within the confines of a clinical MRI system (**A**). A laser displacement system, used only outside of the MRI, allowed for the measurement of leg motion across multiple loading cycles (**B**). Straps across the thigh and shin were used to restrict concomitant motion of the knee (flexed at ~10°) under compression, permitting a sagittal slice through the undeformed and deformed knee tissues to be imaged during cyclic loading (**C**). Although the cartilage can be imaged before and during loading, the measurement of nominal measures does not provide internal information, and there is insufficient image texture for digital image correlation techniques. Because dualMRI is based on phase contrast, internal displacements can be measured at each imaged pixel, providing intratissue deformations not otherwise measurable.

A double-acting pneumatic cylinder in the MRI-compatible loading device was used to apply and release load to the legs of subjects (Fig. 2A). Laboratory-based experiments were performed outside of the MRI system to track the motion of the foot with respect to a static component on the device with a laser displacement system. These displacements showed that the foot position during the load plateau had a maximum standard deviation of 93 μm (Fig. 2B). Analysis of the motion of the foot within each loading cycle showed that an average creep of up to 131 μm occurred during the load plateau, which was 1.4 s in duration (Fig. 2B). Therefore, for *in vivo* loading of the human leg with this leg-loading apparatus, 262 μm was defined as the finest spatial resolution of imaging that was acceptable to image the tibiofemoral cartilage based on previously defined criteria for quasi-steady state load-displacement response across loading cycles and allowable creep within a loading cycle^35^.

### Displacement and Strain Precisions

**Figure 3:**
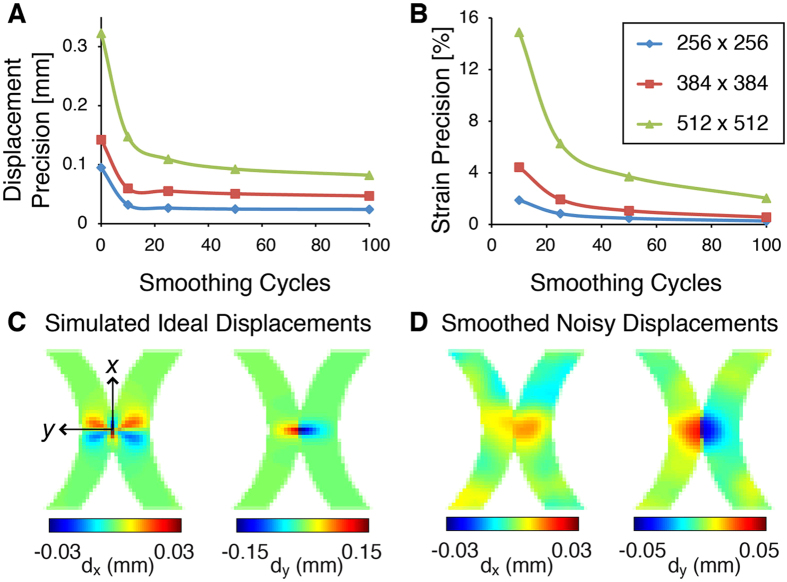
Displacement and strain–precision and effect of smoothing on displacement fields. dualMRI experiments of a silicone imaging phantom were repeated at three different spatial resolutions to permit the calculation of displacement (**A**) and strain (**B**) precision. Precision improved with smoothing at all spatial resolutions but leveled off with less smoothing at coarser spatial resolutions, which also provided higher signal-to-noise ratios. Noise was added to simulated displacements of idealized cartilage-cartilage contact (**C**) to test the effect of smoothing in thin cartilage geometries. Displacement fields retained key features despite a general “flattening” of displacement gradients that is expected with smoothing (**D**).

Prior to measurements in humans, repeated dualMRI experiments of a cyclically loaded silicone gel phantom were used to estimate the precision, or repeatability, of displacement and strain measures^33^,^36^,^37^. The size of the acquisition (i.e. pixel) matrix was varied as a primary image parameter because it directly affects the spatial resolution when the field of view is fixed. Displacement precision, prior to any smoothing, was 95, 142, and 323 μm when the acquisition matrix was 256 × 256, 384 × 384, and 512 × 512 pixels, respectively (Fig. 3A). Smoothing^38^ improved displacement precision to 24, 47, and 82 μm and also permitted the calculation of strain precisions, which were respectively 0.3%, 0.6%, and 2.0% after 100 cycles of smoothing (Fig. 3B). Based on these results and analysis (further detailed in the *Methods* and *Discussion*), an acquisition matrix of 384 pixels × 384 pixels was chosen for *in vivo* imaging.

Under these acquisition parameters, experiment-based noise added to simulated displacements (Fig. 3C) showed that displacement and strain error (5.2 μm and 0.2%, respectively, after 100 smoothing cycles) remained less than the experimental precision. The smoothing parameters used in this study permitted the visualization of prominent displacement patterns in the thin cartilage even after added noise and smoothing (Fig. 3D).

### *In Vivo* dualMRI of Human Knee Cartilage

**Figure 4:**
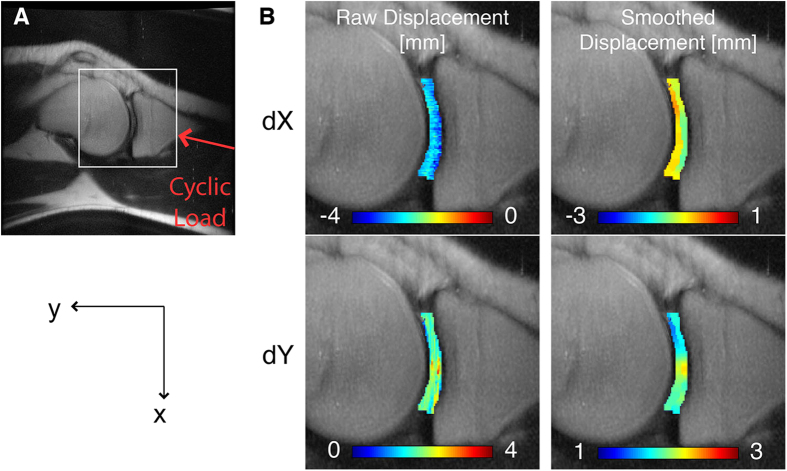
Intratissue displacement fields in tibiofemoral articular cartilage. dualMRI was used to measure *in vivo* displacements under cyclic loading and within a sagittal slice through the medial compartment of the knee (**A**) for a representative male subject. Displacements in the loading direction (y) and the direction transverse to loading (x) showed both rigid body displacement and intratissue deformation (**B**).

Five female and five male volunteers with no medical history of cartilage or knee problems were recruited for *in vivo* dualMRI; one female was disqualified from the study due to mild claustrophobia at the time of imaging. Age, height, weight, and compressive force applied at the foot (equal to half the body weight of each subject), were recorded for subjects imaged in this study (Table 1). It is worth noting that due to restraints and friction on subjects, the load transferred to the knee was likely smaller than this value. Tissue morphology was imaged before and during cyclic loading (Fig. 2C) to confirm that joint tissues were responding to the applied loads. In-plane displacements and strains were quantified using dualMRI within the femoral and tibial cartilage, in a sagittal plane through the medial compartment and overlapping the centroid of the cartilage-cartilage contact area (Fig. 4A). The predominant displacements of cartilage ROIs (i.e., rigid body motion) indicated both compression and shear between the femur and tibia (Fig. 4B). Average velocities within the ROIs during loading were also computed by dividing displacements by the mixing time (Supplementary Figure S1).

**Figure 5:**
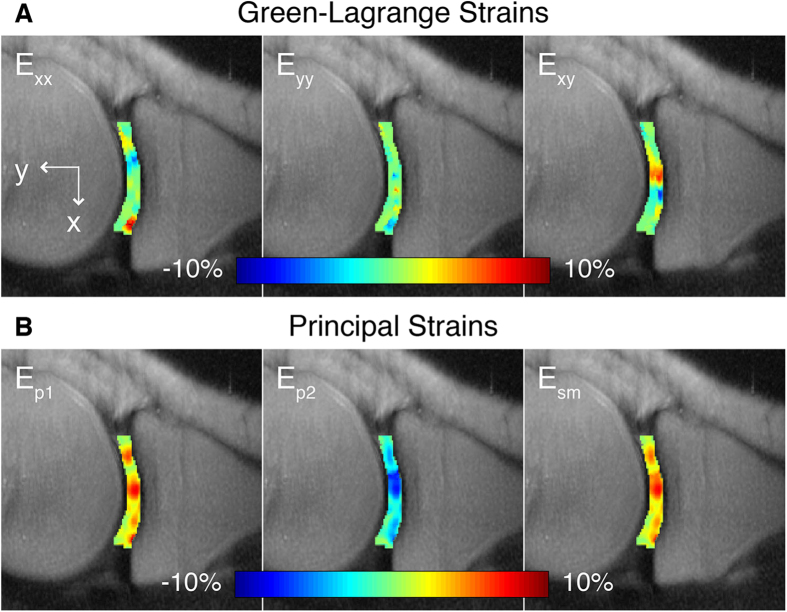
Complex finite strains in the load-based Cartesian coordinate system and principal directions. After smoothing of displacement fields, in-plane Green-Lagrange strains (*E*_*xx*_, *E*_*yy*_, *E*_*xy*_) could be computed at each pixel in the femoral and tibial cartilage (**A**). *E*_*xy*_ was greatest at the middle of the cartilage-cartilage contact region. Computation of the principal strains (*E*_*p1*_, *E*_*p2*_) and maximum shear strains (*E*_*sm*_) for each pixel in the ROIs also showed that these values were the greatest at the middle of the cartilage-cartilage contact region (**B**).

The heterogeneous strain fields within the cartilage ROIs were used to visualize the intratissue deformation under cyclic loading (Fig. 5). Congruent to the compressive and shearing rigid body motions, the strains in the loading direction and shear strains showed the highest magnitudes. The magnitude and direction of highest tensile strain visually corresponded to the load-bearing region of the cartilage-cartilage contact area within the imaged volume (Fig. 5B). Intratissue strains were estimated based on smoothed displacements and averaged across tibial and femoral cartilage ROIs (Table 2).

**Figure 6:**
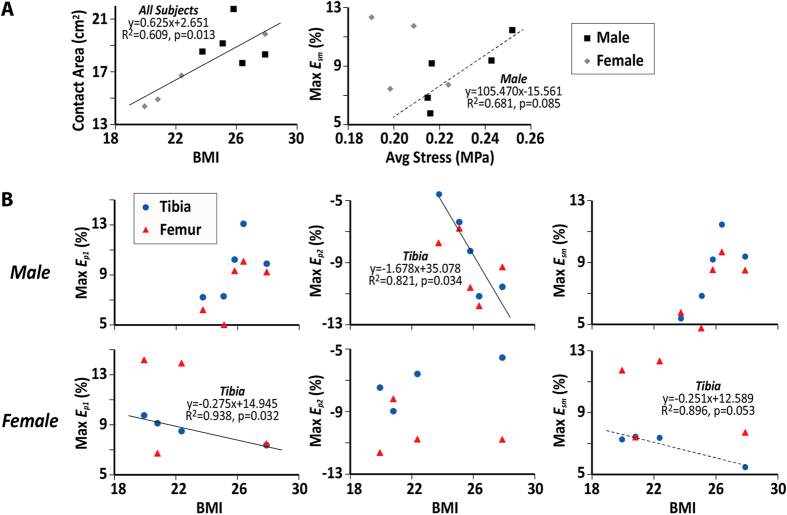
Greater body mass index and estimated average cartilage stress correspond to larger strains. BMI was linearly correlated, in the limited sample size, with contact area for all subjects (**A**) maximum *E*_*p2*_ in the tibia of males (**B**) and maximum *E*_*p1*_ and *E*_*sm*_ in the tibia of females (**B**). Maximum *E*_*sm*_ (**A**) also increased in magnitude with the average stress in the cartilage of one knee during two-legged standing, which was calculated as half the body weight divided by the estimated cartilage contact area. Solid regression line indicates p < 0.05; dashed line indicates p < 0.10.

To consider some effects of geometry, the cartilage contact areas were also measured from MRI anatomical images (Fig. 6A). Strains were compared to the estimated average stress during standing on both feet, which was defined as half the body weight divided by cartilage contact area (Fig. 6A). The maximum *E*_*sm*_ increased with estimated average stress in males but not females. To compare strains among different subjects, the maximum value of principal strains (*E*_*p1*_ and *Ε*_*p2*_) and *E*_*sm*_ in the ROI were compared to the body mass index (BMI) (Fig. 6B). Higher BMI corresponded to greater cartilage strains in the tibia of males (p = 0.034 for maximum *E*_*p2*_, *n* = 5), but smaller cartilage strains in the tibia of females (p = 0.032 for maximum *E*_*p1*_ and 0.054 for maximum *E*_*sm*_, *n* = 4).

**Figure 7:**
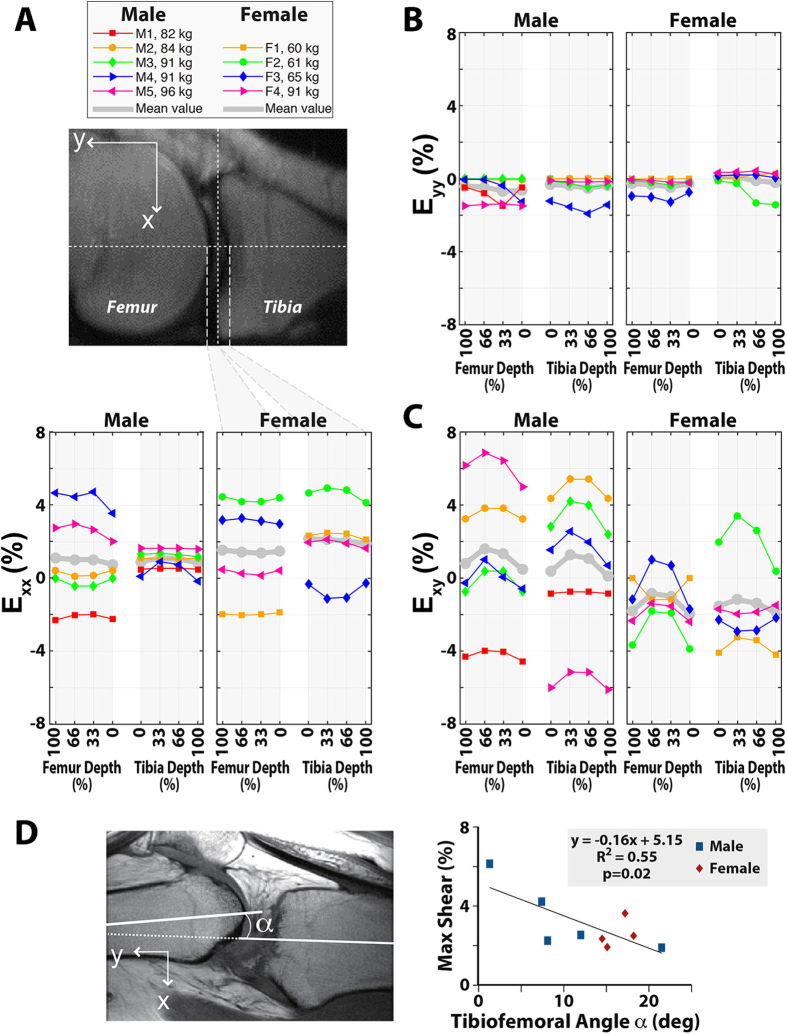
Depth dependent strains in the femur and tibia. Interpolated strain data show the relationship between cartilage depth and *E*_*xx*_ (**A**) E_yy_ (**B**) and *E*_*xy*_ (**C**) for each subject (thin lines), as well as the mean for male and female groups (thick line). These strains show a large variation among male and female subjects in *E*_*xx*_ and *E*_*xy*_. Some of this variation may be explained by the differences in the tibiofemoral flexion angle of the subjects (**D**).

To examine the relationship between strain and tissue depth, femoral and tibial strains were interpolated to compare values at each quartile of the cartilage depth (Fig. 7). To analyze the effects of knee posture, all strains were plotted in Cartesian coordinates in which the *y* axis aligned with the loading direction. A large variation was seen in shear strain (*E*_*xy*_) as well as transverse strain (*E*_*xx*_), which may be due to inter-subject variations in constraining the leg against concomitant motion. The averaged results also showed that the maximum strain in the loading direction (*E*_*yy*_) was in the middle thickness of the cartilage, corresponding with previously reported intratissue strains^39^. It is important to note that the displacement smoothing process within such a thin ROI is challenging and may affect the depth dependent results. In addition, to investigate the effects from the posture and knee motion on the strain results, the tibiofemoral flexion angle (α) was measured (Fig. 7D) and corresponded to variations in *E*_*xy*_. Higher initial flexion angle, which results on greater dissipation of the axial load applied at the foot, corresponded to a smaller *E*_*xy*_. The depth-dependency of *E*_*xx*_ and *E*_*xy*_ varied between subjects, suggesting the need to improve the standardization of knee flexion angle and restriction of concomitant motions of the knee during cyclic loading.

**Figure 8:**
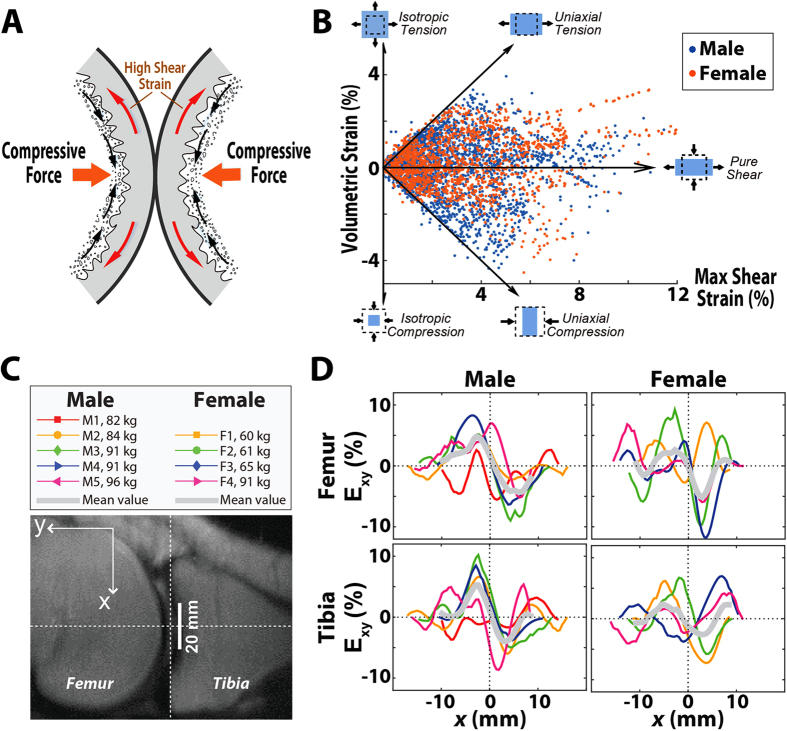
Patterns of shear strain in cartilage. Large shear strains are generated by the contact of convex cartilage surfaces bound to rigid bony substrates (**A**). Volumetric strain was plotted against maximum in-plane shear strain (*E*_*xy*_) for each pixel in the cartilage ROIs of all subjects (**B**). Five different strain states of “isotropic tension,” “uniaxial tension,” “pure shear,” “uniaxial compression,” and “isotropic compression” were delineated with arrows, with the majority of the pixels more aligned with conditions of pure shear. Patterns of *E*_*xy*_ as a function of anterior-posterior position (**C**) with respect to the center of the contact region (*x* = 0) were graphed for the femoral and tibial cartilage of male and female subjects (**D**).

We found that amplitudes of *E*_*xy*_ were generally greater than *E*_*yy*_ in all subjects. Due to the incompressible properties of cartilage under relatively rapid loading (see *Discussion*) and the constraints from stiff underlying bone, the shear strain can be larger than strain in the loading direction^40^ (Fig. 8A; Supplementary Figure S2). A comparison of volumetric strain and *E*_*sm*_ throughout the cartilage ROIs showed that shear strain is the dominant state *in vivo* for tibiofemoral cartilage during compressive loading at the foot (Fig. 8B). Although the spatial pattern of *E*_*xy*_ varied among subjects, strains averaged across subjects exhibited a common trend, especially for male subjects (Fig. 8C,D). Additionally, cartilage posterior to the center of the cartilage-cartilage contact (dashed line) showed a positive shear strain, while *E*_*xy*_ was negative anterior of the center of contact, indicating that tissues are shearing away from the contact area.

## Discussion

In this study, we measured with noninvasive dualMRI the in-plane displacement and strain within the tibiofemoral cartilage of human volunteers. To our knowledge, this is the first *in vivo* study of its kind to directly measure the intratissue strains at pixel/voxel locations within human knee cartilage, which overcomes challenges of quantifying small motions under physiologically-relevant conditions of a thin, high-stiffness tissue embedded deep within the joint space. For *in vivo* experiments, we used imaging parameters optimized to our loading system and tissue of interest, establishing the necessary framework for future studies using this technique. Data from human volunteers revealed concentrated strain patterns at cartilage contact areas and high shear strains due to the incompressibility of cartilage during relatively rapid loading.

The average displacement of cartilage ROIs can be related to the overall rigid body motion of the bone underlying the cartilage, which in turn relates to the joint kinematics under the cyclic loading applied from the foot towards the hip. The predominant motions of the cartilage ROIs indicate that, although compression only was applied at the foot, the cartilage of the knee linkage experienced both compression (superior-inferior) and shear (anterior-posterior) between the femur and tibia (Fig. 4B).

Corresponding to these compressive and shearing rigid body motions, *in vivo* strains in the loading direction and shear strains within the imaged sagittal plane showed high magnitudes, leading to the choice of *E*_*p2*_ and *E*_*sm*_ as fiduciaries for the overall strain environment in the cartilage ROIs. Strains were estimated based on smoothed displacements (Figs 5 and 6, Table 2) and corresponded to strains calculated using computational models based on *in vivo* fluoroscopy and MRI^41^. As expected and also corroborated by MRI studies of changes to cartilage thickness under loading^42^,^43^, strain magnitudes within the femur and tibia were similar during cyclic loading. Knowledge of the internal deformation and strains can lead to an understanding of the biomechanical environment experienced by the cells at the tissue and extracellular matrix length scales, revealing possible linkages to mechanotransductive events, including shear-induced expression of lubricating proteoglycans^44^.

Additionally, comparison of body weights and cartilage contact areas to maximum in-plane strains showed that, as expected, larger cartilage contact areas corresponded to subjects with greater BMI in all subjects and males with greater average stress (Fig. 6A). The BMI also resulted in greater cartilage strains in some measures for males or females (Fig. 6B), with the large variation in strains likely due to subject-to-subject differences in factors such as joint geometry, including condyle size, and possibly also other factors like cartilage composition (e.g. proteoglycan content). Interestingly, the female with the highest body weight (90.7 kg) and BMI (27.9) showed a relatively low strain compared with other females, which may be related to the significant correlation of cartilage surface area to BMI^45^. We expect that a larger sample size will better elucidate relationships between strain and BMI for males and females throughout the joint cartilage.

The differences in strain dependent relationships between the males and females in these analyses were unexpected and surprising. Differences may be due, in part, simply to subject variation in the small sample size studied thus far. The differences may also be attributed to variation in BMI, gender-related differences in cartilage surface and volume^45^, or the larger femur-tibia angle. Interestingly, compared to male subjects, female subjects in this study had on average larger differences in strain magnitudes between the femur and tibia (Table 2). This phenomenon may correspond to the greater flexion angle (Fig. 7D) and smaller femoral condyle size of females^46^, which leads to a strain concentration in the femoral cartilage. Future studies designed with larger subject cohorts will be required to distinguish among these and other explanations for the male-female differences observed in this study.

The large variation in *E*_*xy*_ and *E*_*xx*_, analyzed along the depth at the contact location (Fig. 7) may be related to concomitant knee motions resulting from different flexion angles. In the axial direction, motion was better restricted and corresponded to smaller variations in *E*_*yy*_. Values for *E*_*xx*_ were mostly positive (Fig. 7A), showing that cartilage expanded laterally due to its incompressibility under rapid loading^40^. Incompressibility of cartilage was further confirmed for our study using the calculation of fluid permeation in cartilage by Darcy’s law^47^, with parameters defined in previous studies from our laboratory^34^. Similar with previous studies^34^,^48^, the permeability coefficient was estimated as *v* = 0.09 μm/s. Considering the mixing time of 600 ms in our study, the water permeation was negligible during the rapid time of loading, and cartilage was therefore considered to be incompressible.

Due to the compliance and incompressibility of cartilage under the compressive loading conditions of our study, cartilage exhibits a large lateral expansion while constrained by the stiff underlying bone (Fig. 8A), giving rise to high shear strains. In all the subjects, the proportion of pixels within the ROIs that experienced large magnitudes of pure shear was high compared to other loading modes (Fig. 8B), meaning that the shear strain played a prominent role in the observed cartilage deformation^40^. To confirm the effects of this expansion, a simple linear elastic finite element model of the setup showed that the maximum *E*_*xy*_ was twice that of the maximum *E*_*xx*_ (Supplementary Figure S2). Similar to the depth dependence of *E*_*xy*_ (Fig. 7C), the distribution of *E*_*xy*_ varied in the anterior-posterior direction. Nonetheless, the average *E*_*xy*_ pattern provided insight into the mechanical behavior of the cartilage across subjects. Shear strain at the center of contact was near zero (Fig. 8A), while the shear strains posterior and anterior to center of contact indicated shearing deformations in opposing directions.

In contrast to *in vitro* studies that quantified the maximum transverse strain at the superficial layer^34^,^49^,^50^, this *in vivo* study showed the strain distribution varying among subjects. Average *E*_*yy*_ (Fig. 7B) was highest at mid-depth within the cartilage, which matches some previous studies(e.g.^34^) while conflicting with others that show that *E*_*yy*_ should be greatest at the articular surface^49^,^50^. It is important to note that the factors influencing the strain pattern included the inter-subject variations in joint geometry and flexion angle, the distribution of cartilage properties, and the mechanical effects of surrounding tissues including menisci and ligaments, which were not directly measured in this study^33^. Other factors include the smoothing process, although smoothing can be reduced in future studies through the acquisition of higher SNR data by signal averaging over additional scan repetitions. Because flexion angle affected the strains (Fig. 7D), dissipation of the load and repeatability of joint motion likely varied between subjects. This may explain the higher variation of strains in transverse direction compared to the axial strains. However, to understand these strain variations better in future work, three-dimensional dualMRI should be utilized to visualize volumetric displacement and strains throughout all soft tissues of the joint.

In addition to describing *in vivo* intratissue displacements and strains within a sagittal plane, this study outlined the strategies for implementing dualMRI in measurement of soft tissue mechanical behavior in any system of interest. Experiments were initially performed outside the MRI system to determine the optimal load and imaging parameters for assessing cartilage displacements and strains. Because the leg consists of a series of viscoelastic segments and tissues in complex relation to each other, achievement of a true steady state response to cyclic loading was not expected. Instead, in order to minimize motion artifacts, a quasi-steady state response to cyclic load was required during the synchronized load plateau and image acquisition. This quasi-steady state response to loading^35^ was defined by the criterion that the standard deviation of displacement that occurs across multiple cycles of loading must be less than one-fourth the spatial resolution of imaging. In addition, the overall creep within a single loading cycle must also be less than half the pixel length. Defined by these constraints, we determined the finest spatial resolution compatible with dualMRI of the *in vivo* knee (262 μm) and used this parameter to guide the selection of other imaging parameters. In studies of different tissues with different loading conditions, similar preliminary studies would be minimally time consuming and are an absolute necessity to best tailor dualMRI to the application.

Likewise, the selection of an appropriate acquisition matrix for the desired field of view must undergo similar refinement, following our experiments comparing the acquisition matrix size and spatial resolution of imaging. Although the largest matrix size permitted the highest spatial resolution and smallest pixels to describe subtle changes in tissue structures, which are both an advantage for cartilage imaging, acquisition of the 512 × 512 matrix required the longest imaging time within each load-imaging cycle. Because the displacement-encoded signal decays with time, a longer acquisition period results in greater signal loss by the time the larger components of the signal are acquired at the echo time. Additionally, at this acquisition size, the precision of displacement measurement before any smoothing (323 μm) was almost the same as the spatial resolution (352 μm), negating the advantage of ability of phase contrast imaging to attain displacement measures below half the pixel length. On the other hand, while the 256 × 256 acquisition had the fastest acquisition time within each load-imaging cycle, the resulting spatial resolution (703 μm) would only be able to achieve ROIs of 3 to 4 pixels deep in healthy adult human cartilage, which is typically 2 to 3 mm thick, and would be more appropriate for larger tissues like the intervertebral disc^30^. Because displacement smoothing for dualMRI data is accomplished with a 5 × 5 pixel Gaussian kernel^51^, the intermediate matrix size (i.e. 384 × 384), which permits at least 5 pixels across the thickest regions of cartilage, was chosen for *in vivo* human cartilage dualMRI.

The choices in image acquisition and processing parameters, especially the spatial resolution, were therefore based on the geometry of the tissue of interest. Because articular cartilage is much thinner than other soft orthopaedic tissues, including meniscus and intervertebral disc, these parameters are likely near the technical limit of dualMRI at 3.0 T. The selection of imaging parameters necessitates compromises among overall imaging time, in- and out-of-plane resolution, displacement encoding, and other image acquisition parameters in achieving an optimal signal-to-noise ratio, which affects the quality of the phase data used to calculate directly the displacements at each pixel. Higher signal intensity at the same spatial resolution, a combination that both improves precision and reduces the amount of smoothing necessary, would possibly require magnetic field strengths higher than 3.0 T in a large-bore MRI system. Accordingly, future studies in different tissues (e.g. intervertebral disc, ligament) should consider the spatial resolution requirements as well as the time constraints associated with both cyclic loading and imaging of living subjects.

As with any developing technology, there are a few limitations to this initial translation to *in vivo* dualMRI in human volunteers. Because displacements were only measured in a single sagittal plane, shear in the medial-lateral direction as well as varus-valgus and internal-external rotation could not be discerned. This limitation was primarily dictated by the total imaging time allowable for the human subjects participating in this study, as adding out-of-plane strains and multiple image slices would increase the total imaging time and therefore may affect subject comfort during the extended period of inactivity. However, additional standard MRI scans before and during loading, acquired in synchrony to cyclic loading, could be used to estimate three-dimensional joint kinematics and determine the coupled motions at the knee under compression of the leg via the foot. As with any complex geometry, partial volume effects may affect the phase data at pixels that contain the tissue border or interface. However, since displacements measured in this phase contrast technique represent the signal-weighted average of all spins (i.e. protons) within the pixel, even with partial volume effects, cartilage spins will dominate those of surrounding tissues because the imaging parameters used herein were tuned to optimize the cartilage signal-to-noise ratio.

The loading apparatus used in this study was also limited by the use of non-magnetic materials and constrained by the dimensions of the standard sized bore in the MRI system. The size of the pneumatic cylinder and responsiveness of the electro-pneumatics system in ramping up to and down from the maximum load limited the magnitude and frequency of the loading. Additionally, the loading apparatus featured only discrete adjustments to accommodate variation in the length of subject legs, rather than a continuous range that could more precisely match a particular volunteer. This design required that fine-tuning of a volunteer’s position be made at the hip and lumbar back in order to reduce motion of the leg under load and achieve a more consistent force transfer. These personalized adjustments that could also have contributed to the variation between subjects as well as the differences in trends and significances of correlations observed between the (taller) male and female groups. Future dualMRI research utilizing *in vivo* cyclic loading of human or animal subjects should endeavor to adjust the loading setup for anthropomorphic variations, match subjects across groups by weight, height, or other physical measures, and normalize intratissue strains by appropriately chosen parameters for intra- and inter-subject comparisons.

This work establishes dualMRI as a new tool for the noninvasive, biomechanical assessment of articular cartilage *in vivo*. Imaging techniques that measure cartilage morphology alone cannot easily determine intratissue deformation^52^ and are not typically sensitive to subtle biomechanical changes with early tissue degeneration^53^,^54^. Here, dualMRI was achieved in thin articular cartilage, likely at or near the technical limits for the technique using conventional equipment at a magnetic field strength of 3 T, and revealed complex deformation throughout the tissue during joint contact. Coupling of noninvasive dualMRI deformation and strain measurements with mathematical relationships could also allow for the estimation of mechanical quantities (e.g. intratissue stress) *in vivo* that are not otherwise possible^4^. From this multi-subject study, the functionality and reliability of dualMRI has been initially verified. We anticipate that dualMRI will provide the musculoskeletal research community with (1) a clinical diagnostic tool to evaluate efficacy of therapeutic agents to target early degeneration in animal and human trials, (2) the ability to functionally evaluate cartilage healing and repair with emerging therapies, (3) baseline data describing the healthy function of human cartilage *in vivo*, and (4) a platform technology to more broadly study biomechanical function of load-bearing soft tissues (e.g. meniscus, ligament, intervertebral disc) *in vivo*.

## Methods

Institutional Review Board approval was obtained from the Human Research Protection Program at Purdue University for all aspects of this study. The methods described were carried out in accordance with the approved guidelines, and informed consent was obtained from all participants.

### Cyclic Loading Apparatus Design

An MRI compatible loading apparatus was manufactured with wooden, plastic, and non-magnetic metal components and an MRI-safe double-acting pneumatic cylinder (Fig. 2A). The cyclic loading regime was controlled by an electro-pneumatic system that has been previously used by our group^33^. One leg of the human subject was positioned so that the foot rests in a rigid plastic support that is directly connected to the pneumatic cylinder. The knee was flexed at ~10°, and the contralateral leg was permitted to relax next to the loaded leg. The backrest was positioned at 25° from horizontal to restrict the horizontal motion of the hip and lumbar in response to the compressive loading. Plastic straps were tightened over the ipsilateral thigh and shin to further constrain large rigid body motions of the leg in response to compression. With the patient and apparatus in the 3.0 T clinical MRI system (General Electric Signa HDx, Waukesha, WI), an 8-channel knee volume radiofrequency coil was positioned around the ipsilateral knee without interference by the loading apparatus, permitting imaging of the knee during cyclic loading.

### Quasi-steady State Load Response

To confirm that repeatable deformation could be applied to knee cartilage, a single human subject (male, 23 years old, 83.9 kg) was tested in the laboratory environment, as described in this section, and also in the clinical MRI system (see *In vivo dualMRI of Human Tibiofemoral Articular Cartilage*). The subject had no medical history of ankle, knee or hip injuries in either the loaded and imaged leg or the contralateral leg. Outside of the MRI system, a laser two-dimensional displacement sensor (Keyence LJ-G080, Woodcliff Lake, NJ) was attached to a fixed location on the load apparatus to measure the motion of the plastic support attached to the pneumatic cylinder. With the hip constrained by the backrest and, in part, by the thigh strap, the laser displacement sensor measured the displacement of the leg in the direction of compression (Fig. 2B). Because a rigid point of reference could not be physically fixed to the bones of the subjects, the displacement of the foot represented the maximum displacement in the foot-knee-hip linkage in the direction of loading and was used as a conservative proxy for the rigid body displacement of the knee joint. Loads of 411 N (50% body weight) were applied every 5.0 s for 100 cycles, and a load plateau (exclusive of ramp up and down of load) was achieved for at least 1.4 s within each loading cycle.

### dualMRI Acquisition and Analysis

Electronic triggering was used to synchronize imaging to the cyclic loading regime. Displacement-encoded MRI was accomplished with an encoding gradient of 0.33 π/mm in each direction of interest, and acquired with a single-shot fast spin echo (ssFSE) acquisition sequence^30^. A mixing time (TM) of 600 ms was used to permit the ramp-up of load prior to image acquisition during the load plateau. Displacements at each pixel were determined from the phase data using custom software (MATLAB, v. 7.10, Mathworks, Natick, MA), as previously described^30^,^33^,^34^. Cartilage ROIs were manually segmented (by LC) using software (ImageJ, NIH, Bethesda, MD), and binary masks were created to represent the femoral and tibial articular cartilage ROIs. Displacements were then smoothed with multiple iterations of a 5 × 5 pixel Gaussian kernel^51^ and used to calculate in-plane Green-Lagrange strains^30^,^34^,^51^,^55^.

### Displacement and Strain Precision

A silicone gel (Sylgard 527, Dow Corning, Elizabethtown, KY) was repeatedly tested with dualMRI to analyze the precision of this technique. A separately validated loading device^33^ was used to compress a 1.5-cm thick imaging phantom within the 3.0 T clinical MRI system to 18.5 N for 1.5 s, every 3.0 s. Spatial resolution was varied by using different acquisition matrix sizes (256 × 256/384 × 384/512 × 512 pixels) while maintaining a fixed slice thickness (3 mm) and field of view (180 × 180 mm). Image acquisition parameters necessarily changed with the varying acquisition matrix size and were as follows: echo time (TE) = 62/72/82 ms respectively, effective repetition time (TR) = 3000 ms, number of averages (NA) = 8. To compute precision, 16 evenly spaced points of interest were selected within the deformed image of the silicone phantom. The pooled standard deviation of the displacement and strain values at these points of interest, across five repeated experimental measures, were defined as the precision^33^,^34^,^36^ and computed as a function of the number of smoothing cycles (Fig. 3A,B).

Simulated deformations were generated using a model of contact between femoral and tibial cartilage ROIs in finite element analysis software (COMSOL Multiphysics v3.2, Burlington, MA) (Fig. 3C). The Monte Carlo method was used to study the propagation of error to strain calculations using MATLAB (Mathworks, Natick, MA) with 100 repeated simulations and randomly distributed noise^51^. Within each simulation, displacement noise was added according to the standard deviation of displacements before smoothing, as determined for the 384 × 384 acquisition matrix, and noisy displacements were smoothed (Fig. 3D) to permit estimation of strain. The displacement and strain bias was computed as the difference between the ideal values and the values after smoothing in 12 evenly distributed points within the ROIs across the 100 simulations, similar to previous studies^51^.

### *In vivo* dualMRI of Human Tibiofemoral Articular Cartilage

Five male and four female subjects (Table 1) were imaged for dualMRI. All imaging sessions were performed by the same operator (DC). Prior to the start of loading within the MRI system, standard anatomical images of the knee (multi-slice gradient echo acquisitions) showed no signs of cartilage or other joint damage and were utilized in the selection of the imaging volumes. All dualMRI images were acquired in a single sagittal imaging plane through the medial compartment of the subject’s right leg, with care taken to select a plane that overlapped with the centroid of the cartilage-cartilage contact area within the compartment. The load parameters were as reported above, and dualMRI, with displacement encoding of 0.33 π/mm and TM of 600 ms, commenced after ~65 cycles of preconditioning. The ssFSE acquisition parameters were echo time = 72 ms, repetition time = 5000 ms, field of view = 180 × 180 mm, slice thickness = 3 mm, and 16 averages. The total imaging time for dualMRI was approximately 35 minutes. Displacements (Fig. 4B) and strains (Fig. 5) were calculated as reported in previous studies^30^,^34^,^51^ and outlined above. For each subject, the cartilage contact area was measured (Fig. 6A), by multiplying the slice thickness with the visible contact length between femur and tibia in all slices^56^,^57^. Cartilage contact pressure was estimated as the piston force, which was set to compressed the foot at one-half body weight, divided by the tibiofemoral contact area, and compared to strains (Fig. 6A). For male and female subjects, the maximum values of *E*_*p1*_, *E*_*p2*_ and *E*_*sm*_ were compared to BMI and average stress (Fig. 6B). Linear regressions were performed to relate strain values with measured quantities, including BMI and average stress.

## Additional Information

**How to cite this article**: Chan, D. D. *et al.* *In vivo* articular cartilage deformation: noninvasive quantification of intratissue strain during joint contact in the human knee. *Sci. Rep.* **6**, 19220; doi: 10.1038/srep19220 (2016).

## Supplementary Material

Supplementary Information

## Figures and Tables

**Figure 1:**
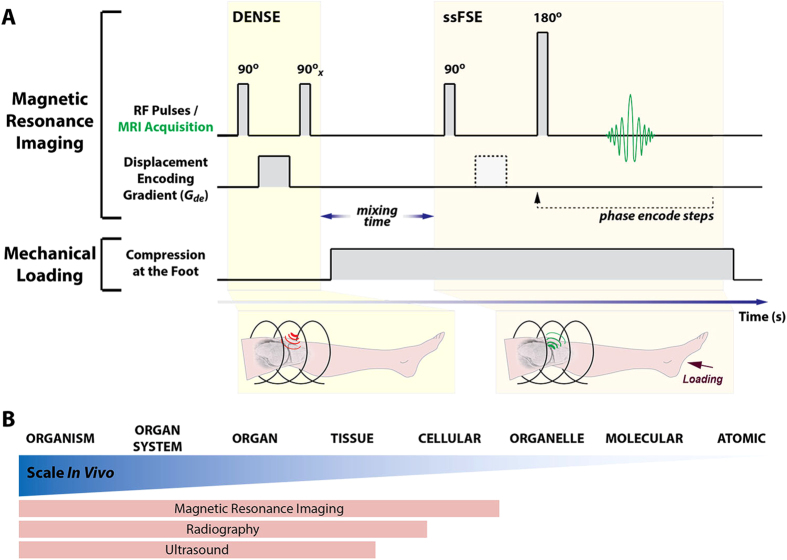
Displacements under applied loading by MRI (dualMRI) and selected techniques for hierarchical characterization of human biomechanics. dualMRI synchronizes magnetic resonance imaging and mechanical loading to reveal intratissue strain (**A**). DENSE (displacement encoding by stimulated echoes) and ssFSE (single shot fast spin echo) enable tracking of the MRI phase signal before and during compressive loading applied to the foot. Medical imaging modalities, like MRI, radiography, and ultrasound, are capable of noninvasive measurement of cartilage biomechanics, and are limited ultimately by the spatial scale for acquisition (**B**). In contrast to other modalities, MRI allows for direct tracking of cartilage displacement and strain at a pixel-by-pixel basis at high spatial and temporal resolution^33^,^58^.

**Table 1:** Basic information about the male (*n* = 5) and female (*n* = 4) volunteers.

	Age (y)	Height (m)	Weight (kg)	Applied Force (N)
Males (*n* = 5)	25.8 (3.0)	1.85 (0.05)	88.6 (5.8)	434.3 (28.6)
Females (*n* = 4)	21.3 (1.7)	1.74 (0.05)	69.3 (14.4)	339.5 (70.7)

The force applied at the foot, through pneumatic actuation, was half of a subject’s body weight (BW). Data is presented as mean (standard deviation).

**Table 2:** Maximum principal strains (*E*_*p1*_, *E*_*p2*_) and shear strain (*E*_*sm*_) in the femur and tibia of males and females.

Principal Strain	Cartilage ROI	Males (*n = 5*)	Females (*n = 4*)
Maximum *E*_*p1*_	Femur	0.080 (0.022)	0.106 (0.040)
	Tibia	0.095 (0.024)	0.087 (0.010)
Minimum *E*_*p2*_	Femur	−0.092 (0.020)	−0.103 (0.015)
	Tibia	−0.081 (0.028)	−0.071 (0.014)
Maximum *E*_*sm*_	Femur	0.074 (0.021)	0.098 (0.026)
	Tibia	0.085 (0.024)	0.069 (0.009)

Strains were measured in the tibial and femoral articular cartilage of volunteers during compressive loading of one-half body weight applied at the foot. Data is presented as mean (standard deviation).
