# Supplementary material for: In vivo articular cartilage deformation: noninvasive quantification of intratissue strain during joint contact in the human knee
Source: Sci Rep. 2016 Jan 11;6:19220. doi: 10.1038/srep19220 (PMC4707486; doi:10.1038/srep19220)
Supplement: Supplementary Information [file srep19220-s1.pdf]

In vivo articular cartilage deformation: noninvasive quantification of intratissue strain during joint contact in the human knee

Deva D. Chan, Luyao Cai, Kent D. Butz, Stephen B. Trippel, Eric A. Nauman, Corey P. Neu

SUPPLEMENTARY INFORMATION

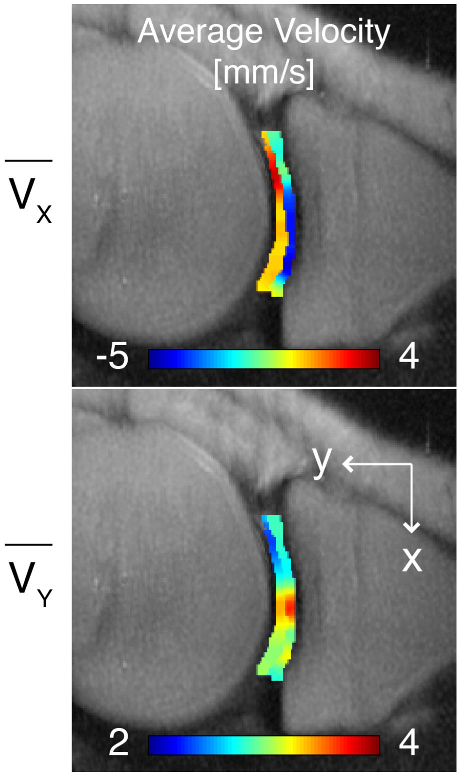

Supplementary Figure S1. Intratissue average velocity in tibiofemoral articular cartilage. The average velocity at each pixel in the femoral and tibial cartilage was also computed in a representative subject using the time elapsed (mixing time) between displacement encoding and image acquisition.

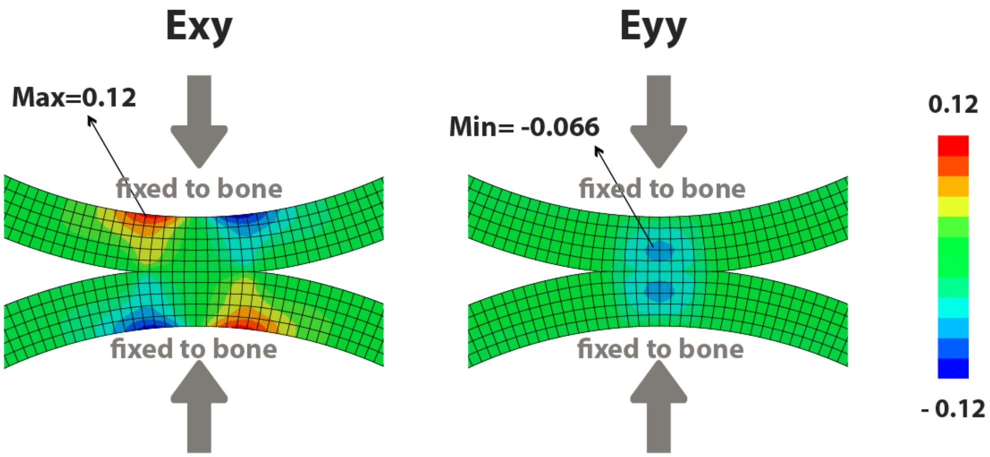

Supplementary Figure S2. E_{xy} and E_{yy} results of an incompressible linear elastic model under axial loading. This simple example represents femur and tibia cartilages that were fixed at their cartilage-bone interface. The result showed that the lateral extension of an incompressible cartilage can induce a larger shear deformation compared to axial deformation.
